# Preoperative iron supplementation in non-anemic patients undergoing major surgery: a systematic review and meta-analysis

**DOI:** 10.1016/j.bjane.2025.844618

**Published:** 2025-04-04

**Authors:** Fabio Vieira Toledo, Daniel De Carli, Jose Fernando Amaral Meletti, Herman Yuri Almeida Togo, Italo Pires Gomes, Renato Makoto Sakashita, Lucas Felix Montes, Rafael Santos Tiburcio, Cesar de Araujo Miranda

**Affiliations:** Faculdade de Medicina de Jundiaí (FMJ), Departamento de Anestesiologia, Jundiaí, SP, Brazil

**Keywords:** Anemia, Blood cells, Blood transfusion, Iron, Perioperative care

## Abstract

**Background:**

Blood transfusions are associated with increased morbidity and mortality, and maintaining global blood supplies can be a challenge. This systematic review investigates the impact of preoperative iron supplementation on the risk of blood transfusion among non-anemic patients undergoing major surgeries.

**Methods:**

We conducted a systematic search of PubMed, Embase, and Cochrane Central for randomized controlled trials published up to May 2024. Studies involving the use of erythropoietin, or patients already using iron supplementation when trial randomization was conducted were excluded. Outcomes assessed included the number of individuals who received blood transfusions, and mean hemoglobin levels at the first day and by the first postoperative week.

**Results:**

A total of 1,162 non-anemic patients from 9 studies were included. Of these, 54% received preoperative iron supplementation. The average age was 71 years, and 44% were women. Preoperative iron supplementation was associated with a significantly lower risk of receiving a blood transfusion (OR = 0.54; 95% CI 0.40 to 0.75; p < 0.001). At the first postoperative day, the iron supplementation group had significantly higher mean hemoglobin levels compared to the no-treatment group (MD = 0.22 g.dL^-1^; 95% CI 0.02 to 0.42; p = 0.03). However, the pooled results could not rule out the null hypothesis for the difference in mean hemoglobin levels throughout the first week (MD = 0.12 g.dL^-1^; 95% CI -0.12 to 0.35; p = 0.34).

**Conclusion:**

Preoperative intravenous iron supplementation in non-anemic patients undergoing major surgeries, particularly cardiac procedures, significantly reduces transfusion requirements. However, the benefits of oral iron remain uncertain, and further research is warranted to establish standardized perioperative supplementation protocols.

**PROSPERO identifier:**

CRD42024552559.

## Introduction

Perioperative anemia is common in patients undergoing major surgeries and is associated with adverse clinical outcomes, such as increased mortality, higher incidence of postoperative complications, and prolonged hospital stays.^1^ The need for blood transfusions during these surgeries is significant and varies widely. Studies indicate that red blood cell concentrates may be necessary for up to 92.8% of patients undergoing cardiac surgeries and 100% of some types of non-cardiac surgeries.^2^, ^3^, ^4^

Moreover, maintaining blood supplies is a challenge. The National Health Service Blood and Transplant (NHSBT) issued a warning in late-2022 due to the risks associated with compromising blood stocks.^5^ This led to the intensified implementation of the Patient Blood Management (PBM) program, aimed at reducing the consumption of blood components, not only to improve outcomes by decreasing the risks inherent in transfusion but also to ensure a blood supply for every patient in need. Strategies include managing preoperative anemia and optimizing hematopoiesis, with iron supplementation being widely studied.^5^^,^^6^

Iron plays a fundamental role in hemoglobin synthesis. Therefore, correcting iron deficiency increases the efficiency of hemoglobin production and oxygen-carrying capacity.^7^ Previous meta-analyses have shown a reduction in the demand for blood transfusions through iron supplementation in surgical patients with anemia.^8^^,^^9^ However, few studies have examined iron supplementation in non-anemic patients undergoing surgeries, such as total knee arthroplasty. Most of them are non-randomized, and the randomized studies often lack the power to detect specific outcomes in non-anemic patients, showing inconsistent results.^10^, ^11^, ^12^, ^13^

Therefore, a comprehensive evaluation focusing solely on non-anemic patients is essential to address these gaps. Given the increasing number of major surgeries performed daily and the critical need to limit transfusions to reduce morbimortality, costs and alleviate pressure on blood supplies, further investigation in this population is warranted. This systematic review aims to evaluate the impact of preoperative iron supplementation on the incidence of blood transfusions and the mean hemoglobin levels on the first postoperative day and at the end of the first postoperative week in non-anemic patients undergoing major surgeries.

## Method

This systematic review and meta-analysis was conducted and reported in accordance with the Cochrane Collaboration's Manual for Systematic Reviews of Interventions and the Preferred Reporting Items for Systematic Reviews and Meta-Analyses (PRISMA) guidelines.^14^^,^^15^ The study protocol was registered in the International Prospective Register of Systematic Reviews (PROSPERO; ID number CRD42024552559).

### Eligibility criteria

We restricted inclusion to the following criteria: (1) Randomized Controlled Trials (RCTs); (2) Comparing any formulation of iron supplementation with placebo or no treatment, initiated in the preoperative period; (3) Among non-anemic adults (≥ 18 years) undergoing major surgery, regardless of the presence of iron deficiency. The absence of anemia was defined as any value above the cut-off determined by the laboratory standards of each study.

We excluded studies with (1) Use of erythropoietin or other erythropoiesis-stimulating agents alone or in combination with iron; (2) Patients who were already taking iron supplementation at the time of randomization in the clinical trial; (3) Performance of autologous whole blood therapy; or (4) Studies with overlapping populations.

### Definition of major surgeries

To define the articles that evaluated major surgeries, the criteria established by the European Surgical Association were used, which classifies a surgery as “major” based on factors such as the complexity of the procedure, the presence of significant comorbidities, vascular clamping or organ ischemia, high intraoperative blood loss, the need for norepinephrine, prolonged operative time, perioperative blood transfusion requirements, a significant systemic inflammatory response, and the need for intensive or intermediate postoperative care.^16^

### Search and data extraction strategy

The search strategy for this systematic review was designed to comprehensively identify relevant studies in PubMed (MEDLINE), Embase, and Cochrane Central Register of Controlled Trials for articles meeting the eligibility criteria published from inception to May 2024. A combination of Boolean operators AND, OR, and specific keywords were applied to create a structured search query. Keywords were grouped into categories to capture studies by surgical procedures, iron supplementation, and clinical trials: (operative OR perioperative OR preoperative OR surgeries OR "Surgical Procedures, Operative"[Mesh] OR surgery OR "surgical procedures" OR "Anesthesia"[Mesh] OR anesthesia OR "Specialties, Surgical"[Mesh] OR "Perioperative Care"[Mesh] OR "Perioperative Period"[Mesh]) AND (“Iron Compounds"[Mesh] OR "iron compounds" OR "Ferric Compounds"[Mesh] OR "ferric compounds" OR "ferrous sulfate" OR "ferric carboxymaltose" OR "ferrous sulphate" OR "iron isomaltoside" OR injectafer OR "iron dextri-maltose" OR ferinject OR "iron therapy" OR "perferryl iron" OR "iron replenishment" OR “iron supplements”) AND (randomized controlled trial[pt] OR controlled clinical trial[pt] OR clinical trials as topic[mesh:noexp] OR trial[ti] OR random*[tiab] OR placebo*[tiab]).

The detailed search strategy used in each database is available in the Supplemental Material (Table S1). No language restriction was applied.

Two authors (F.T. and H.T.) independently performed the literature search following predefined search criteria and resolved discrepancies by consensus. Baseline characteristics and outcome data were independently extracted by two authors (C.M. and F.T.). A template was developed for data extraction of relevant items, including study details (first author, publication year, sample size of non-anemic patients, type of surgery, route of administration), participants’ baseline characteristics (age, sex), intervention, control, and outcome measures. Disagreements were also resolved by consensus among the authors. Five corresponding authors were contacted for additional data, and three provided the information.

### Endpoint and subgroup analysis

The primary outcome was the number of patients undergoing at least one allogeneic blood transfusion. Secondary outcomes included the mean hemoglobin levels at the first day and by the end of the first postoperative week.

Both primary and secondary outcomes were reanalyzed by stratifying studies into subgroups of either oral or Intravenous (IV) iron supplementation, in order to explore differences between the two routes of administration. For the primary outcome, an exploratory assessment was also conducted, categorizing patients between those undergoing cardiac and non-cardiac surgeries.

### Sensitivity analyses

We performed the leave-one-out sensitivity analysis for the primary outcome to assess the effects of influential studies on the pooled analysis and overall heterogeneity. Studies were sequentially removed, and data were reanalyzed to ensure the stability of the pooled effects. Publication bias for the primary outcome was examined through a Begg's funnel plot to evaluate the symmetric distribution of trials with similar weights and Egger's regression asymmetry test. Finally, in the subgroup receiving oral iron, we performed a meta-regression of the impact of treatment duration on the effect measure. Within this subgroup, we also conducted a leave-one-out analysis to investigate any changes in the effect measure with the exclusion of a study with less than two weeks of oral therapy.

### Risk of bias assessment

Two independent authors (F.T. and H.T.) appraised the risk of bias using the Cochrane Collaboration's Risk of Bias Assessment Tool (RoB-2) for RCTs. Disagreements were resolved through consensus. In cases where consensus could not be reached, a third author (C.M.) was consulted to adjudicate the decision. This tool judged the risk of bias as high, some, or low in each of five domains: randomization process, deviations from intended interventions, missing outcome data, measurement of the outcome, and selection of the reported result.^17^

To assess the level of certainty of the evidence for each outcome, two authors employed the Grading of Recommendations, Assessment, Development and Evaluation (GRADE) tool using the GRADEpro Guideline Development Tool.

### Statistical analysis

We pooled Odds Ratios (OR) and Mean Differences (MD) with 95% Confidence Intervals (95% CI) for categorical and continuous outcomes, respectively. A p-value less than 0.05 was considered significant. DerSimonian and Laird random-effects models were employed for all outcomes, due to expected differences in the populations and methods of each study. Heterogeneity was assessed using the I² statistic and Cochran's *Q* test; p < 0.10 and I² > 25% were considered significant for heterogeneity.

Review Manager 5.4 (Nordic Cochrane Centre, The Cochrane Collaboration, Denmark) and R-Studio, version 4.4.0 (R Foundation for Statistical Computing), were used for the statistical analysis.^18^^,^^19^

### Trial sequential analysis

In order to assess the cumulative effect of iron supplementation on transfusion odds, we performed a post hoc Trial Sequential Analysis (TSA) to evaluate the robustness of the evidence for the primary outcome and for the cardiac versus non-cardiac subgroups. An α level of 5%, a power of 80%, and the observed odds ratio reduction were used. We used a random effect model, due to potential heterogeneity. We conducted this analysis using the TSA software (Copenhagen Trial Unit, Centre for Clinical Intervention Research, Copenhagen).^20^

## Results

### Study selection and baseline characteristics

**Figure 1:**
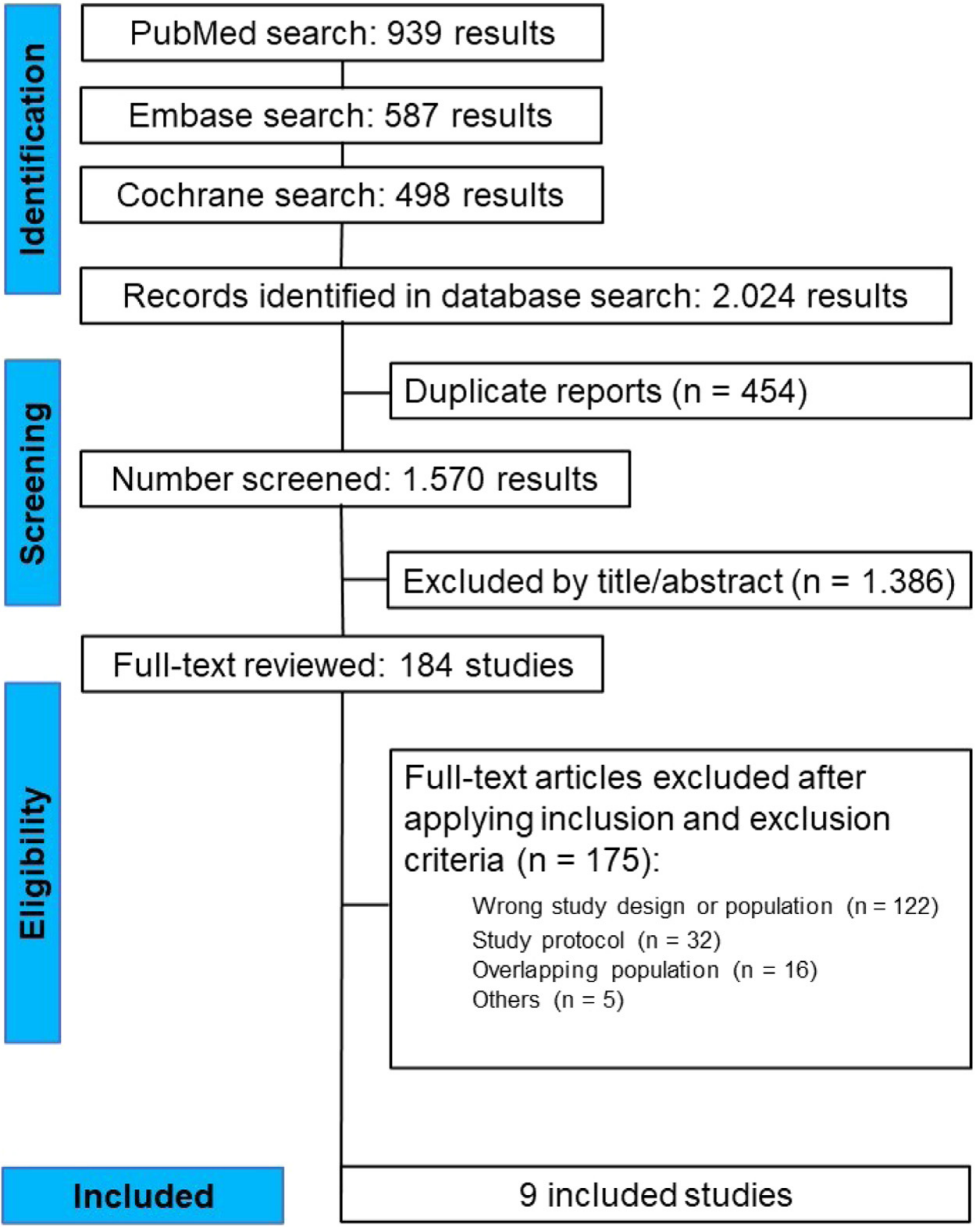
PRISMA flow diagram of screening and selection of studies.

The initial search yielded 2024 articles on May 30, 2024. After removing 454 duplicate results and excluding 1386 studies based on title and abstract screening, 184 studies were selected for full-text review, as detailed in Figure 1. Of these, 9 studies (8 RCTs and 1 post-hoc analysis of an RCT) fulfilled the pre-specified eligibility criteria and were included in this systematic review and meta-analysis.^11^, ^12^, ^13^^,^^21^, ^22^, ^23^, ^24^, ^25^, ^26^ The main reasons for exclusion were studies with anemic patients only or the initiation of iron therapy intra/post-operatively. No study was excluded solely for not involving major surgeries, that is, all studies that fulfilled the design, intervention, and population criteria were judged unanimously by the reviewers as encompassing major surgeries. Furthermore, no studies involving laparoscopic or robotic surgery were found. A total of 1162 patients were included, of whom 627 (54%) received iron preoperatively. The average age was 71 years, and 44% were women. Half of the included studies used oral iron, while the other half employed IV iron. Overall, baseline characteristics were comparable between groups. Anemia cut-off, iron administration route, and transfusion criteria varied among included studies, as depicted in Table 1. The studies were conducted in Europe (8 studies) and in Western Asia (1 study).

**Table 1:** Baseline characteristics of the population in the included studies.

Study	N^o^ of NoA patients Iron/ Control	Age (years) Iron/ Control	Patients NoA/T	Women Iron/ Control (%)	Surgery	Route and Intervention	Dose of iron^k^	Time to initiate preop therapy	NoA definition (g.dL^-1^)	Transfusion trigger (g.dL^-1^)	Primary outcomes	Follow-up
Serrano-Trenas 2011	60/50	83.5 /82.5^a^	110/ 196	79 (80)/ 77 (79)^a^	Hip fracture	IV/ Iron sucrose	200 mg qod for 6 days	At admission and preop	≥ 12	< 8/ < 9 or symptoms^j^	N° patients transfused	30 days after discharge
Moppett, 2019	23/26	81.2/ 82.5^a^	49/80	24 (62)/ 29 (71)^a^	Hip fracture	IV/ Iron sucrose	200 mg qd for 3 days	1‒3 d preop	≥ 12	< 8 or < 10 with symptoms	Reticulocyte counts on day 7	30 days
Briguglio, 2020	32/35	67.7/ 65.9	67/73	19 (59)/ 20 (57)	Elective hip or knee arthroplasty	Oral/ Iron sucrosomial^b^	30 mg qd	30 days preop	≥ 13M, ≥ 12F	NA	Change in Hb	Few days
Bielza, 2021	74/73	87/87^a^	147 /253	95(75)/ 89(70)^a^	Hip fracture	IV/ Iron sucrose	200 mg qod for 6 days	At admission and preop	≥ 13M, ≥ 12F	< 8 or < 10 with symptoms	Functional gain (Barthel Index)	365 days
Briguglio, 2023	27/24	69.9/ 72.6	51/58	18 (66)/ 15 (62)	Elective hip or knee arthroplasty	Oral/ Iron saccharate^c^	30 mg qd	45 ± 15 days preop	≥ 13M, ≥ 12F^f^	< 10^g^	Change in Hb	±3 days
Lidder, 2007	17/8	68.7/ 71^a^	25/45	5 (29)/ 3 (38)	Surgical colorectal cancer patients	Oral/ Ferrous sulphate	40 mg qd	14 d preop	≥ 13.5M, ≥ 11.5F	< 8 or < 10 with symptoms	Change in Hb	Until hospital discharge
Garrido-Martín, 2012	54 (IV)/ 53 (Oral)/ 52	65/ 65/ 65	159/ 159	16 (30)/ 15 (28)/ 12 (23)	Elective on-pump cardiac surgery	IV/ Iron sucrose OR Oral/ Ferrous fumarate^e^	100 mg qd for 3 days (IV) OR 105 mg qd (Oral)^e^	5‒6 d preop	≥ 13M, ≥ 12F	< 7 or < 8 with symptoms^i^	Change in Hb	30 days after discharge
Weltert, 2023	185/ 169	66/70^a^	354/ 594	111 (36)/ 123 (43)^a^	Elective cardiac surgery	Oral/ Iron sucrosomial^d^	60 mg qd	30 d preop	≥ 13	< 7 or symptoms	Change in Hb	4 days
Friedman, 2023	102/ 98	62.5/ 62.7	200/ 200	10 (10)/ 15 (15)	Elective or urgent on-pump single cardiac surgery (CABG or single valve replacement)	IV/ Ferric carboxymaltose	1000 mg single dose	1‒3 d preop	≥13M, ≥12F	< 8^h^	N° patients transfused	42 days

N°, Number; NoA, Non-anemic; T, Total; Preop, Preoperative; Postop, Postoperative; NA, Not Available; Hb, Hemoglobin; CABG, Coronary Artery Bypass Graft; M, Male; F, Female; qd, Daily; qod, Every other day.

a Demographic data of the general study population.

b Contains ferric pyrophosphate plus vit C.

c Iron saccharate and multivitamins (vit B2, B6, B9, B12, C, E).

d Contains ferric pyrophosphate and multivitamins (vit B12, C) and folic acid.

e Two interventions groups (oral and IV).

f WHO criterion previously used in another study by the same author (Briguglio 2020).

g Transfusion evaluation if < 10.

h And the decision to transfuse is at the attending physicians’ discretion.

i Low output syndrome associated with Hb level < 8 g.dL^-1^ in coronary patients or < 7 g.dL^-1^ in valve surgery patients.

j < 8 or 9 g.dL^-1^ in patients with a history of cardiorespiratory conditions, or any Hb in patients with symptoms of untreated anemia.

k The oral doses were converted to the corresponding elemental iron.

### Pooled analysis

#### Primary endpoint

**Figure 2:**
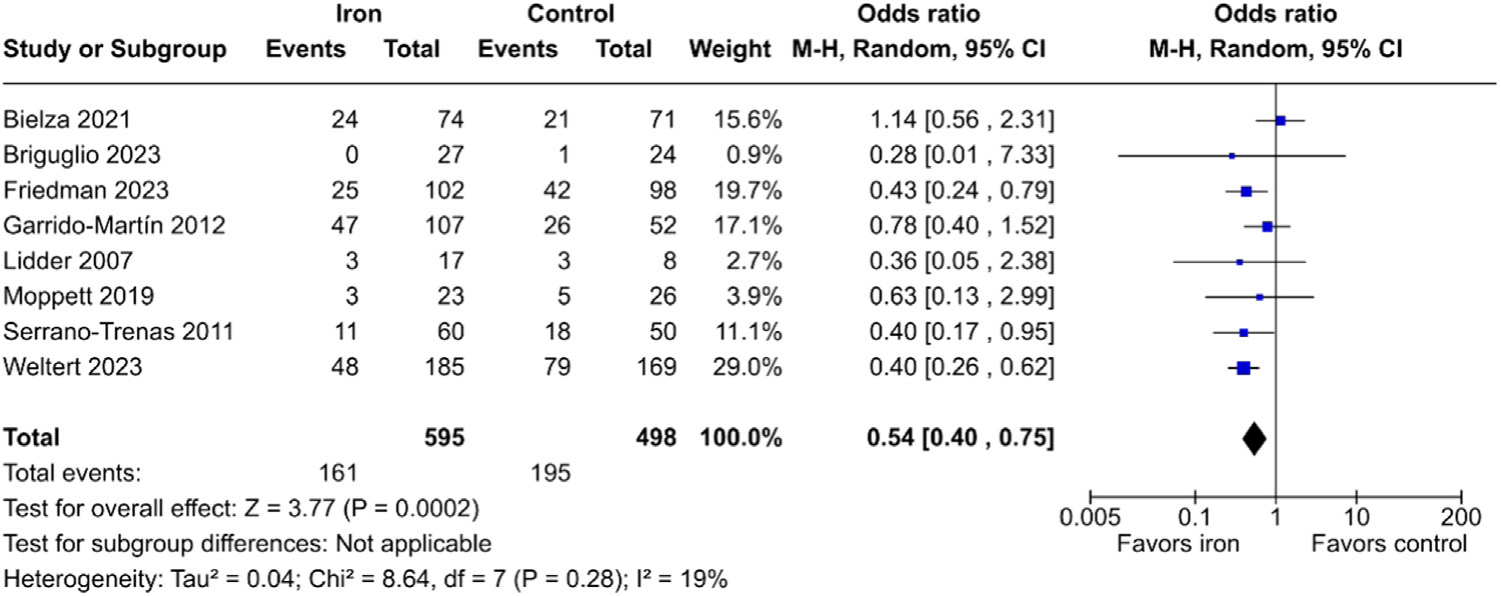
Incidence of blood transfusion in patients with preoperative oral or intravenous iron supplementation compared to controls.

In the pooled analysis for the primary endpoint, comprising 8 studies (n = 1093), the odds of undergoing blood transfusion in patients who received preoperative iron supplementation were 46% lower when compared to those who did not (OR = 0.54; 95% CI 0.40 to 0.75; p < 0.001; I² = 19%; Fig. 2).^11^, ^12^, ^13^^,^^21^, ^22^, ^23^, ^24^, ^25^

#### Primary endpoint sensitivity analysis

The leave-one-out sensitivity analysis for the primary outcome revealed consistent results favoring the iron supplementation group after omitting each individual study (Supplementary Fig. S1). The exclusion of the single study with a high risk^25^ of bias also presented findings consistent with the original results.

In the analysis categorizing studies into those conducted on cardiac versus non-cardiac surgeries, it was observed that, in non-cardiac surgeries, the pooled results could not reject the null hypothesis regarding the odds of blood transfusion with iron supplementation (OR = 0.67; 95% CI 0.40 to 1.14; p = 0.14; I² = 6%).^13^^,^^21^^,^^23^^,^^24^ However, in cardiac surgeries, there was a significant reduction (OR = 0.49; 95% CI 0.33 to 0.72; p = 0.0003; I² = 30%) (Supplementary Fig. S2).^11^^,^^12^^,^^25^ Overall, the test for differences between subgroups was non-significant (p = 0.33).

A TSA was then conducted on the total population and the two subgroups. The results indicate that sufficient sample size was achieved in both the total and cardiac populations, with both TSA analyses crossing the conventional boundary and supporting statistical significance. Conversely, the non-cardiac population did not reach the estimated sample size of 1559. These results are reported in Figures S3, S4, and S5 of the Supplementary Material.

### Secondary endpoints

**Figure 3:**
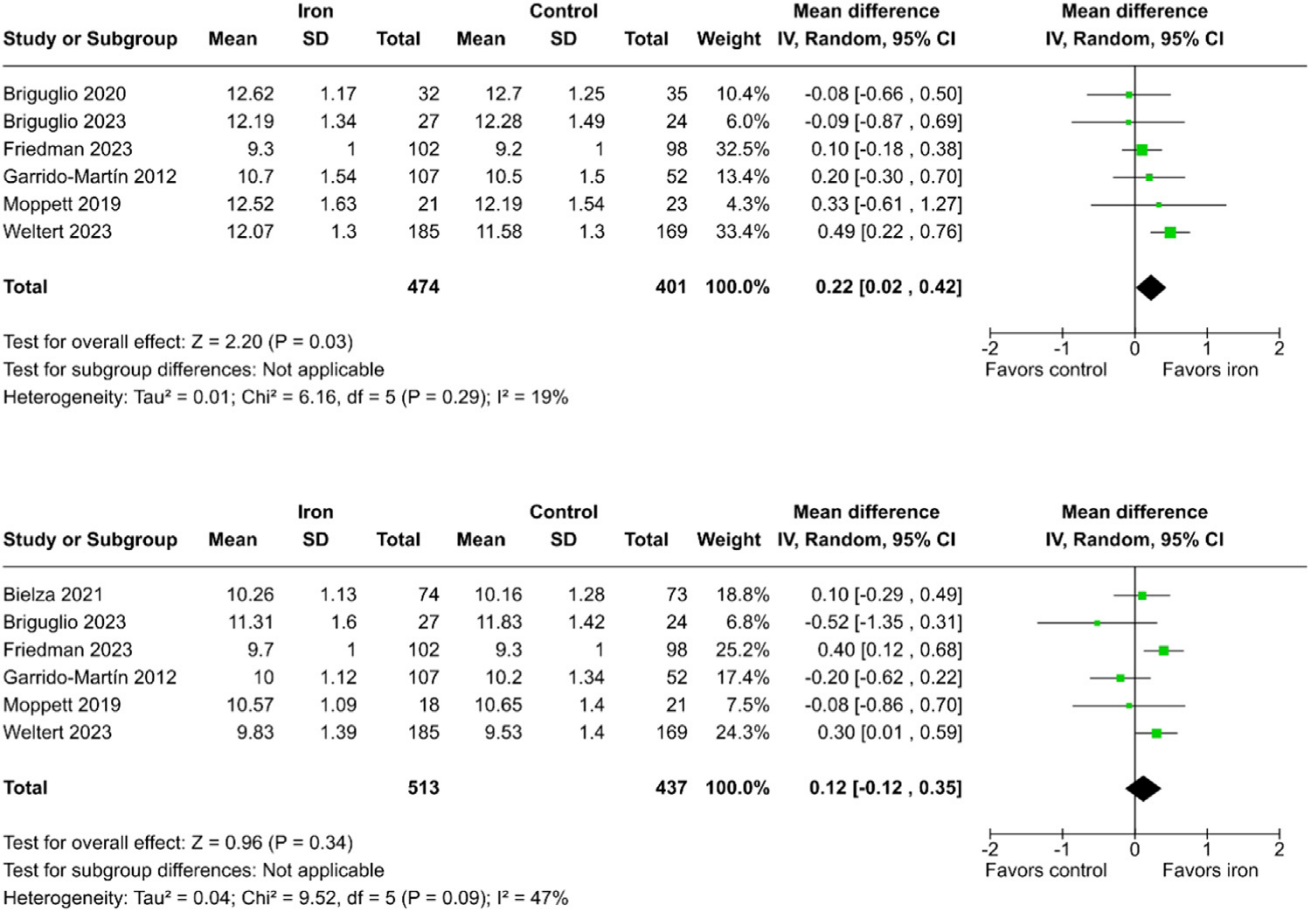
Mean hemoglobin levels on the first postoperative day (a) and first postoperative week (b) in patients who received preoperative oral or intravenous iron supplementation compared to controls.

Iron supplementation led to a significant increase in mean hemoglobin levels at the first postoperative day compared to no supplementation (MD = 0.22 g.dL^-1^; 95% CI 0.02 to 0.42; p = 0.03; I² = 19%; Fig. 3a).^11^, ^12^, ^13^^,^^23^^,^^25^^,^^26^ Nevertheless, it was not possible to reject the null hypothesis regarding the mean hemoglobin levels by the end of the first postoperative week (MD = 0.12 g.dL^-1^; 95% CI -0.12 to 0.35; p = 0.34; I² = 47%; Fig. 3b).^11^, ^12^, ^13^^,^^21^^,^^23^^,^^25^

### Subgroup analysis

#### Oral administration of iron supplementation

**Figure 4:**
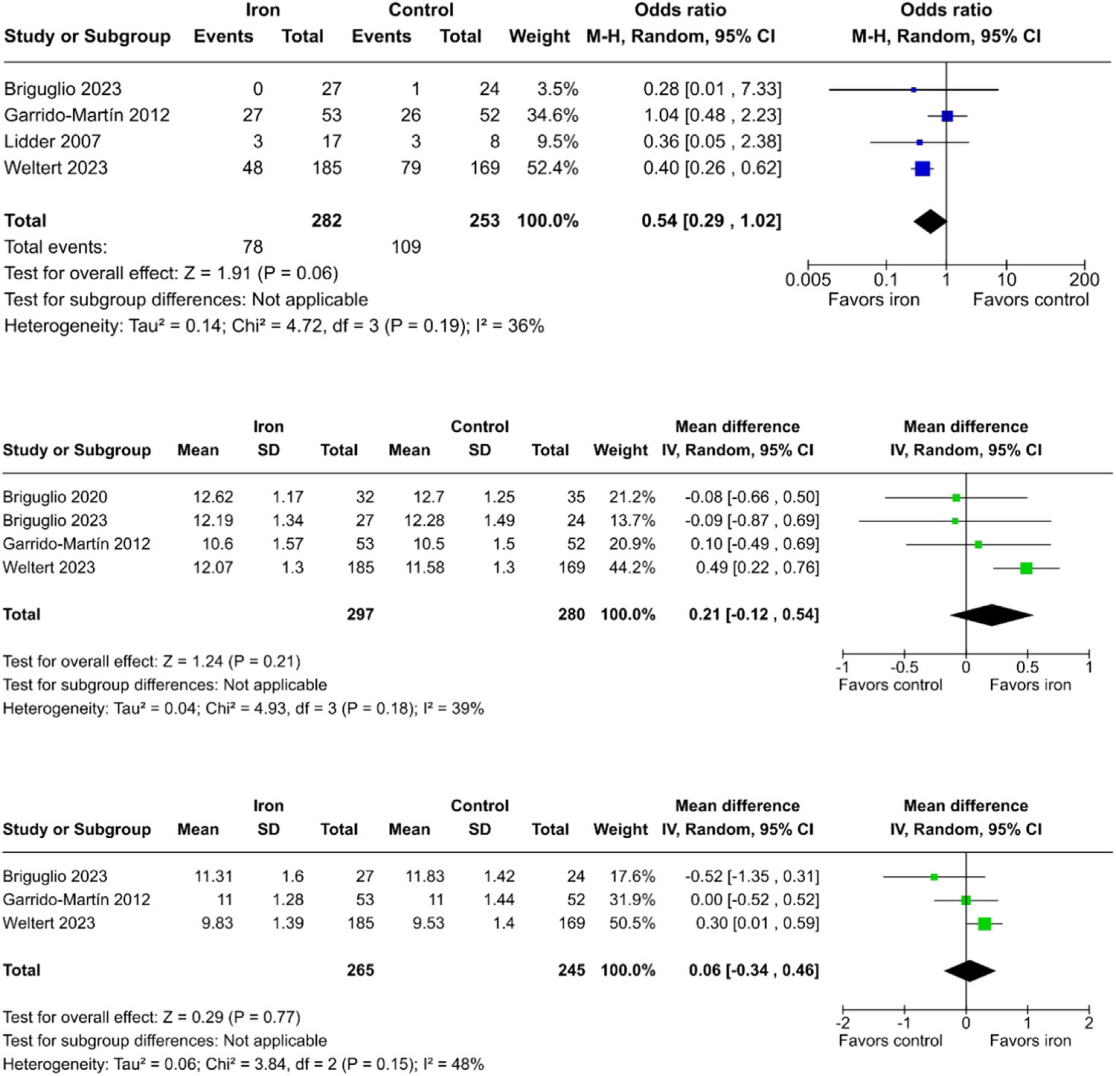
Incidence of blood transfusion (a), mean hemoglobin levels on the first postoperative day (b), and first postoperative week (c) in patients who received preoperative oral iron supplementation compared to controls.

When restricting the analysis to studies that used oral iron supplementation, it was not possible to reject the null hypothesis regarding transfusion rates (OR = 0.54; 95% CI 0.29 to 1.02; p = 0.06; I² = 36%; Fig. 4a);^12^^,^^13^^,^^22^^,^^25^ mean hemoglobin levels at the first postoperative day (MD = 0.21 g.dL^-1^; 95% CI -0.12 to 0.54; p = 0.21; I² = 39%; Fig. 4b);^12^^,^^13^^,^^25^^,^^26^ and mean hemoglobin levels by the end of the first postoperative week (MD = 0.06 g.dL^-1^; 95% CI -0.34 to 0.46; p = 0.77; I² = 48%; Fig. 4c) between the groups.^12^^,^^13^^,^^25^

#### Sensitivity analysis of the oral admin subgroup

The meta-regression based on the duration of preoperative supplementation in the oral subgroup revealed a significant and inversely proportional association between preoperative treatment duration and the treatment effect on odds of undergoing blood transfusion (p = 0.04; Supplementary Fig. S6). In the leave-one-out sensitivity analysis of oral iron administration, the odds of transfusion were statistically lower with the removal of Garrido-Martin et al.^12^ (Supplementary Fig. S7), the study with a shorter duration of oral iron supplementation.

#### IV administration of iron supplementation

**Figure 5:**
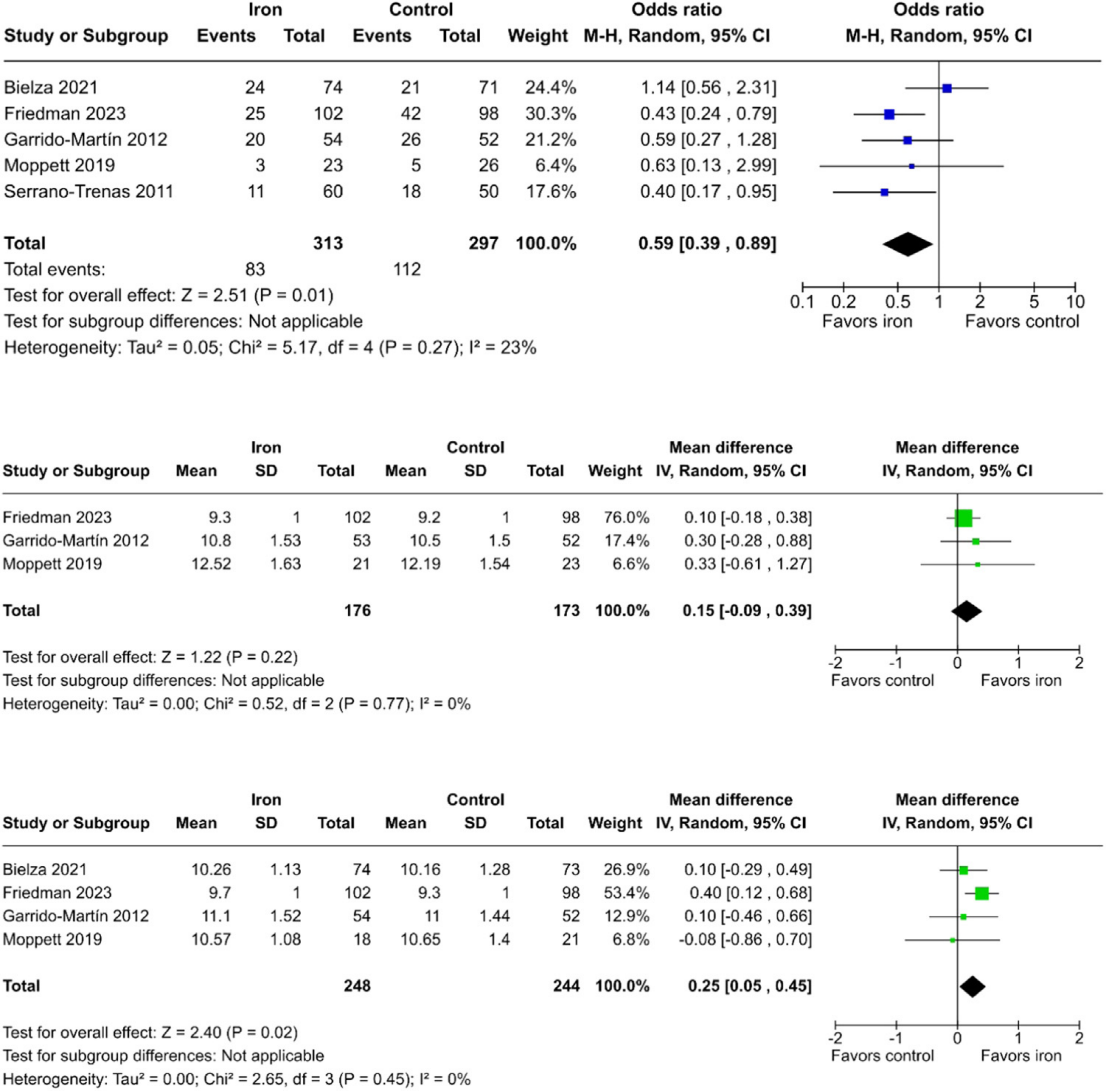
Incidence of blood transfusion (a), mean hemoglobin levels on the first postoperative day (b), and first postoperative week (c) in patients who received preoperative intravenous iron supplementation compared to controls.

When restricting the analysis to studies employing IV iron supplementation, the odds of undergoing blood transfusion in patients who received IV iron were 41% lower when compared to those who did not (OR = 0.59; 95% CI 0.39 to 0.89; p = 0.01; I² = 23%; Fig. 5a).^11^^,^^12^^,^^21^^,^^23^^,^^24^ We could not reject the null hypothesis that the mean hemoglobin levels at the first postoperative day do not differ between groups (MD = 0.15 g.dL^-1^; 95% CI -0.09 to 0.39; p = 0.22; I² = 0%; Fig. 5b).^11^^,^^12^^,^^23^ However, IV iron supplementation led to a significant increase in mean hemoglobin levels by the end of the first postoperative week compared to no supplementation (MD = 0.25 g.dL^-1^; 95% CI 0.05 to 0.45; p = 0.02; I² = 0%; Fig. 5c).^11^^,^^12^^,^^21^^,^^23^

### Risk of bias assessment

The risk of bias analysis using the RoB-2 tool revealed the following results: 5 studies rated as “low risk” in all domains;^11^^,^^12^^,^^21^, ^22^, ^23^ 3 were rated with “some concerns”,^13^^,^^24^^,^^26^ all due to deviations from the intended intervention, with two of them raising additional concerns: one related to the randomization process and the other due to missing outcome data; and 1 rated as “high risk”^25^ resulting from bias in the randomization process (Supplementary Fig. S8). All studies classified with more than minimal risk of bias administered oral iron supplementation. Among these, those classified as “some concerns” belong to the non-cardiac surgery subgroup, while the single one classified as “high risk” belongs to the cardiac surgery subgroup.

There was no evidence of publication bias in the visual analysis of the funnel plot for the transfusion odds outcome, confirmed by Egger's test (p = 0.89) (Supplementary Fig. S9).^27^

### Quality of evidence

According to the GRADE assessment, there was moderate certainty of evidence for transfusion odds and mean hemoglobin at the first postoperative day. Moreover, there was low certainty of evidence for the mean hemoglobin level by the end of the first postoperative week. The certainty of evidence was downgraded due to concerns about the risk of bias in certain studies. The outcome for mean hemoglobin level in the first week was also downgraded due to the imprecision of the outcome, with a wide CI (Supplementary Table S2).

## Discussion

This systematic review and meta-analysis of 9 studies and 1162 patients compared iron supplementation with placebo or no treatment initiated in the preoperative period in non-anemic patients undergoing orthopedic surgeries of the knee and hip, cardiac surgery, and colorectal surgery. The main findings regarding iron supplementation therapy were: (1) Lower incidence of perioperative transfusion; (2) Higher mean hemoglobin levels at the first postoperative day, without statistically significant improvement by the end of the first postoperative week; and (3) Statistically significant benefit of iron supplementation in IV form relative to no iron replacement in this population, in contrast to no significant effect in the oral iron replacement group.

Although implementing this intervention may present challenges and complexities, both due to logistical difficulties and the need for awareness among the professionals involved, the 46% reduction in the odds of requiring a blood transfusion among patients who underwent iron supplementation should not be overlooked, and it supports the potential adoption of this therapy. This is further endorsed by the current medical literature on transfusion risks, which states that transfusions are associated with increased perioperative morbidity and mortality in this population.^2^^,^^3^

This meta-analysis evidenced that IV iron supplementation is associated with 41% lower transfusion rates, while oral supplementation did not reach statistical significance to detect potential benefit. This may also be related to treatment duration. Oral administration is recommended for at least 2- to 4-weeks,^1^^,^^28^ and, in one study, the duration was 5- to 6-days.^12^ In the leave-one-out sensitivity analysis of the oral subgroup, the exclusion of this study resulted in a significantly lower risk of blood transfusion (p < 0.001) (Supplementary Fig. S7). The importance of an adequate treatment duration became even more evident with the meta-regression analysis of the oral subgroup for the number of preoperative treatment days, which showed a significant association between longer treatment durations and increased treatment efficacy, with the OR reduced by 0.035 (p = 0.04) for each added day of treatment (Supplementary Fig. S6).

Hepcidin is an important factor in the regulation of iron absorption, distribution, and storage. Its levels increase secondary to high doses of iron, which reduces enteral absorption to prevent overload, thereby decreasing the effectiveness of oral therapy while increasing side effects such as dyspepsia or constipation.^29^ For this reason, oral iron typically requires longer treatments durations with lower doses compared to IV therapy. Moreover, the metabolic and inflammatory impact of major surgeries disrupts iron regulation, resembling chronic anemia to some extent.^30^ Postoperative drops in serum iron can occur even with adequate stores, characterizing functional iron deficiency linked to elevated Interleukin-6 (IL-6) and C-reactive protein levels.^31^ This inflammatory response increases hepcidin, inhibiting gastrointestinal iron absorption and its release from macrophages and hepatocytes, and reducing availability for erythropoiesis despite normal or high ferritin but low transferrin saturation and serum iron.^32^ Studies indicate that perioperative IV iron can bypass the hepcidin-mediated blockade and prevent functional iron deficiency.^33^

It is important to highlight the considerable gap in the literature regarding the therapeutic regimen for iron replacement, with no consensus on the ideal dosage, for either oral or IV administration. Furthermore, existing protocols focus on the treatment of previously diagnosed iron deficiency.^34^^,^^35^ However, the included studies provided supplementation to non-anemics patients, regardless of iron status evaluation, who underwent major surgeries. The best therapeutic approach for this scenario is still uncertain, and there are no specific PBM guidelines on this matter. Thus, it is expected to detect heterogeneity in the dosing regimens used. This dissent was evident when analyzing the therapeutic regimens used in the selected studies, where widely varying doses were observed: some studies administered 30 mg to 105 mg of elemental iron orally while others used between 100 mg to 1000 mg intravenously.

Pathological factors may also interfere with iron absorption, such as colorectal cancer. The underlying inflammatory process in these conditions can impair oral iron absorption, leading to anemia of chronic disease. This was exemplified in the study by Lidder et al.,^22^ where no statistically significant reduction in transfusion requirements was observed in patients who received oral treatment 14 days prior to colorectal cancer surgery. These patients often respond poorly to oral treatment, and IV therapy may be preferred among patients with anemia of chronic disease.

The effectiveness of IV therapy in reducing the need for transfusion becomes evident when we analyze that most studies employing this method did so for emergency surgeries, whereas oral treatment was used exclusively for elective surgeries. It is well-established that emergency surgeries are an independent risk factor for increased transfusion requirements, with studies indicating a threefold higher risk of receiving a blood transfusion compared to elective procedures.^1^

Another important aspect to highlight is that, although the present meta-analysis evaluated major surgeries with potential risks of bleeding and blood transfusions, cardiac surgeries have specific characteristics that increase these risks, such as hemodilution, coagulopathies, and an intense systemic inflammatory response caused by cardiopulmonary bypass.^36^ Our exploratory analysis categorizing the primary outcome by cardiac vs. non-cardiac surgeries demonstrated that iron supplementation significantly reduced the risk of transfusion in cardiac surgeries (p = 0.0003), while this reduction was not observed in the non-cardiac surgery subgroup (p = 0.14). However, the test for subgroup differences showed no statistical significance, suggesting that the non-cardiac group should probably follow the trend of the cardiac one, at some point showing significant benefits of iron supplementation; hence, it is reasonable to report a combined effect between the subgroups.

We tested this hypothesis more thoroughly with a TSA, which endorsed that the sample size of the non-cardiac surgery subgroup is still underpowered to detect group differences (Fig. S5). Nevertheless, the non-cardiac subgroup's cumulative Z-curve shows a trend toward the superiority zone for iron therapy, crossing it at times, which is consistent with the trend observed in the categorized analysis suggesting that both groups most likely follow the same direction, favoring iron supplementation.

It is worth noting that the three studies evaluating the population undergoing cardiac surgeries had a much greater weight in the combined analysis for blood transfusions compared to the sum of all studies that evaluated patients undergoing non-cardiac surgeries. This point is consistent with the findings from Weltert et al.,^25^ which is a post-hoc analysis of a large randomized clinical trial conducted in cardiac surgeries, previously published.^37^ This study accounted for a substantial portion of our meta-analysis, representing 29% of the total weight in the primary transfusion outcome (Fig. 2) and nearly 45% in the cardiac surgery subgroup (Supplementary Fig. S2). Considering that, in Weltert et al., iron supplementation was performed orally in the preoperative period, these significant results reinforce the idea that iron supplementation can have a robust impact in the context of cardiac surgeries and are in line with our findings.

The lower incidence of perioperative transfusion observed with iron supplementation in our study can be explained by the high prevalence of iron deficiency without anemia in the general population.^38^^,^^39^ Additionally, blood loss during the surgical procedure may lead to increased consumption and, ultimately, depletion of iron stores. Thus, preoperative iron replacement therapy can be equally effective for non-anemic patients with iron deficiency and those who will develop iron deficiency anemia postoperatively.

Since nutritional deficiency is the leading cause of anemia, it is crucial to consider socioeconomic aspects in this analysis. In this meta-analysis, all included studies were conducted in developed countries, where fewer patients with nutritional deficiencies are expected compared to developing countries.^4^^,^^5^ Advanced age is another significant risk factor, as it is associated not only with a decline in marrow function, which increases the risk of anemia, but also a higher likelihood of inadequate protein intake and, consequently, a greater risk of these patients having depleted iron stores.^4^^,^^5^ In addition, the aging process is associated with high concentrations of inflammatory mediators such as IL-6 which, as previously mentioned, interfere with the use of stored iron.^40^ In this review, the average age was 71 years, which might be one of the reasons why iron supplementation significantly reduced the risk of transfusion.

Regarding hemoglobin levels assessed at the first postoperative day, the iron group had a mean value of 0.22 g.dL^-1^ higher than the control group (p = 0.03), while the same assessment one week after surgery showed no statistical difference (p = 0.34). However, it is important to emphasize that the assessment of iron supplementation's impact on erythropoiesis in the postoperative period is significantly affected by blood transfusion, which may introduce confounding in the association between postoperative hemoglobin levels and iron supplementation. Postoperative blood transfusion may tend to equalize hemoglobin levels between groups, suggesting that this equivalence was achieved at the expense of higher transfusion rates in the control group.

The difficulty in establishing strict and uniform transfusion criteria that allow for comparisons across different studies can be partly explained by the fact that, according to the PBM program, anemia tolerance should be individualized and not solely based on predefined transfusion triggers.^41^^,^^42^ Nevertheless, although based on individualized approaches, the studies included in this meta-analysis had quite homogeneous definitions for the transfusion triggers. That is, all studies respected the concept of tailoring decisions to each patient's clinical profile (Table 1). In addition, most of the studies randomized and blinded the control and intervention groups, ensuring that the decision whether or not to transfuse followed predefined criteria, without knowledge of the group to which the patient belonged.

This study has limitations. First, there was noticeable heterogeneity in iron supplementation practices observed in the analyzed studies. This variability included differences in the duration of supplementation, types of iron used, dosages, routes of administration and timing of hemoglobin assessment. In addition, there was variability in the anemia definitions in each study. However, the leave-one-out sensitivity analysis and TSA maintained consistent results for the primary outcome (Figs. S1 and S3). To strengthen the evidence and establish practical guidelines for preoperative iron supplementation, future clinical trials with sufficient power to detect differences in this patient group are warranted, especially more studies in other major surgical settings, such as major spine and oncological surgeries. Additionally, future studies should adopt homogeneously controlled approaches including the selection of hard primary outcomes.^43^

## Conclusions

In conclusion, this systematic review and meta-analysis found that preoperative iron supplementation in non-anemic patients undergoing major surgeries reduced the need for postoperative blood transfusion, particularly in patients undergoing cardiac surgeries and among those who received IV iron supplementation. It was also observed that preoperative iron supplementation improved hemoglobin levels at the first postoperative day. The role of oral iron remains uncertain and warrants further study. Future large-scale, well-powered trials are needed to refine perioperative iron supplementation protocols, particularly in non-cardiac surgical populations.

## Authors’ contributions

Conception and design of the study: F.T.

Supervision: F.T., D.C., C.M.

Data extraction: F.T., D.C., C.M., H.T., J.M.

Data analysis and interpretation: All authors.

Drafting the original manuscript: All authors.

Revising subsequent drafts: All authors.

Approved the final version of the article to be published: All authors.

## Funding

This research did not receive any specific grant from funding agencies in the public, commercial, or not-for-profit sectors.

## Conflicts of interest

The authors declare no conflicts of interest.

## Appendix Supplementary materials

Image, application 1

## Footnotes

Supplementary material associated with this article can be found in the online version at doi:10.1016/j.bjane.2025.844618.
