# Supplementary material for: Preoperative iron supplementation in non-anemic patients undergoing major surgery: a systematic review and meta-analysis
Source: Braz J Anesthesiol. 2025 Apr 4;75(3):844618. doi: 10.1016/j.bjane.2025.844618 (PMC12053702; doi:10.1016/j.bjane.2025.844618)
Supplement: Supplementary file 1 [file mmc1.docx]

**BJAN-D-24-00637_ Supplementary Materials**

**Supplementary Table S1** Search strategy.

| **Articles were searched using the following keywords.** | |
| --- | --- |
| **PubMed; search on May, 2024** | |
| **Search** | **Terms** |
| #1 | (operative OR perioperative OR preoperative OR surgeries OR "Surgical Procedures, Operative"[Mesh] OR surgery OR "surgical procedures" OR "Anesthesia"[Mesh] OR anesthesia OR "Specialties, Surgical"[Mesh] OR "Perioperative Care"[Mesh] OR "Perioperative Period"[Mesh]) |
| #2 | ("Iron Compounds"[Mesh] OR "iron compounds" OR "Ferric Compounds"[Mesh] OR "ferric compounds" OR "ferrous sulfate" OR "ferric carboxymaltose" OR "ferrous sulphate" OR "iron isomaltoside" OR injectafer OR "iron dextri-maltose" OR ferinject OR "iron therapy" OR "perferryl iron" OR "iron replenishment" OR “iron supplements”) |
| #3 | (randomized controlled trial[pt] OR controlled clinical trial[pt] OR clinical trials as topic[mesh:noexp] OR trial[ti] OR random*[tiab] OR placebo*[tiab]) |
| #4 | #1 AND #2 AND #3 |
| **Cochrane; search on May, 2024** | |
| **Search** | **Terms** |
| #1 | (operative OR surgery OR "surgical procedures" OR anesthesia OR "Perioperative Care" OR "Perioperative Period" OR perioperative OR preoperative OR surgeries) |
| #2 | ("iron compounds" OR "ferric compounds" OR "ferrous sulfate" OR "ferric carboxymaltose" OR "ferrous sulphate" OR "iron isomaltoside" OR injectafer OR "iron dextri-maltose" OR ferinject OR "iron therapy" OR "perferryl iron" OR "iron replenishment" OR “iron supplements”) |
| #3 | #1 AND #2 |
| **Embase; search on May, 2024** | |
| **Search** | **Terms** |
| #1 | (operative OR perioperative OR preoperative OR surgeries OR 'surgery'/exp OR surgery OR "surgical procedures" OR 'surgery'/exp OR 'anesthesia'/exp OR anesthesia OR 'perioperative care'/exp OR 'perioperative period'/exp) |
| #2 | ('iron compounds therapeutic use'/exp OR 'iron compounds administration'/exp OR "iron compounds" OR 'ferric ion'/exp OR "ferric compounds" OR "ferrous sulfate" OR "ferric carboxymaltose" OR "ferrous sulphate" OR "iron isomaltoside" OR injectafer OR "iron dextri-maltose" OR ferinject OR "iron therapy" OR "perferryl iron" OR "iron replenishment" OR “iron supplements”) |
| #3 | ('controlled clinical trial'/exp OR (random* OR placebo*):ti,ab OR trial:ti) AND [embase]/lim |
| #4 | #1 AND #2 AND #3 |

**Supplementary Table S2** Grading of Recommendations, Assessment, Development and Evaluation (GRADE).

| **Certainty assessment** | | | | | | | **Nº of patients** | | **Effect** | | **Certainty** | **Importance** |
| --- | --- | --- | --- | --- | --- | --- | --- | --- | --- | --- | --- | --- |
| **Nº of studies** | **Study design** | **Risk of bias** | **Inconsistency** | **Indirectness** | **Imprecision** | **Other considerations** |  |  | **Relative (95% CI)** | **Absolute (95% CI)** |  |  |
| **Transfusion** | | | | | | | | | | | | |
| 8 | Randomized trials | Serious^a^ | Not serious | Not serious | Not serious | None | 161/595 (27.0%) | 195/498 (39.1%) | **OR 0.54** (0.40 to 0.75) |  | ⨁⨁⨁◯ Moderate | Critical |
| **First day hemoglobin** | | | | | | | | | | | | |
| 6 | Randomized trials | Serious^b^ | Not serious | Not serious | Not serious | None | 474 | 401 | ‒ | Mean **0.22 g.dL^-1^ more** (0.02 to 0.42 more) | ⨁⨁⨁◯ Moderate | Critical |
| **First week hemoglobin** | | | | | | | | | | | | |
| 6 | Randomized trials | Serious^c^ | Not serious | Not serious | Seriousᵈ | None | 513 | 437 | ‒ | MD **0.12 g.dL^-1^ higher** (0.12 lower to 0.35 higher) | ⨁⨁◯◯ Low | CRITICAL |

CI, Confidence Interval; MD, Mean Difference; OR, Odds Ratio.

^a^ 2 studies “some concerns” and 1 study “high”.

^b^ 2 studies “some concerns” and 1 study “high”.

^c^ 1 study “some concerns” and 1 study “high”.

^d^ The optimal information size criterion is met, and the 95% Confidence Intervals overlaps no effect.

**Supplementary Figure S1** Leave-one-out sensitivity analysis: Number of patients transfused in total.


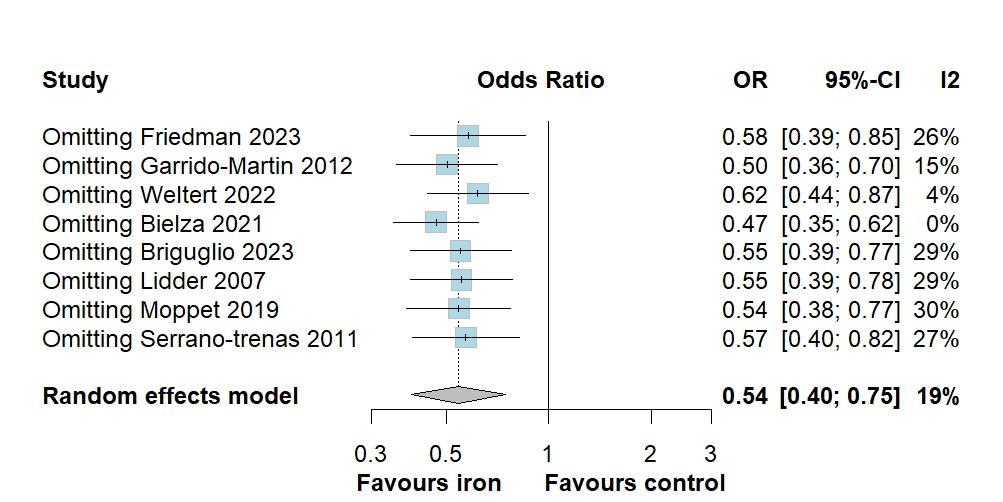


**Supplementary Figure S2** Forest plot for analysis of categorization into cardiac versus non-cardiac subgroups.


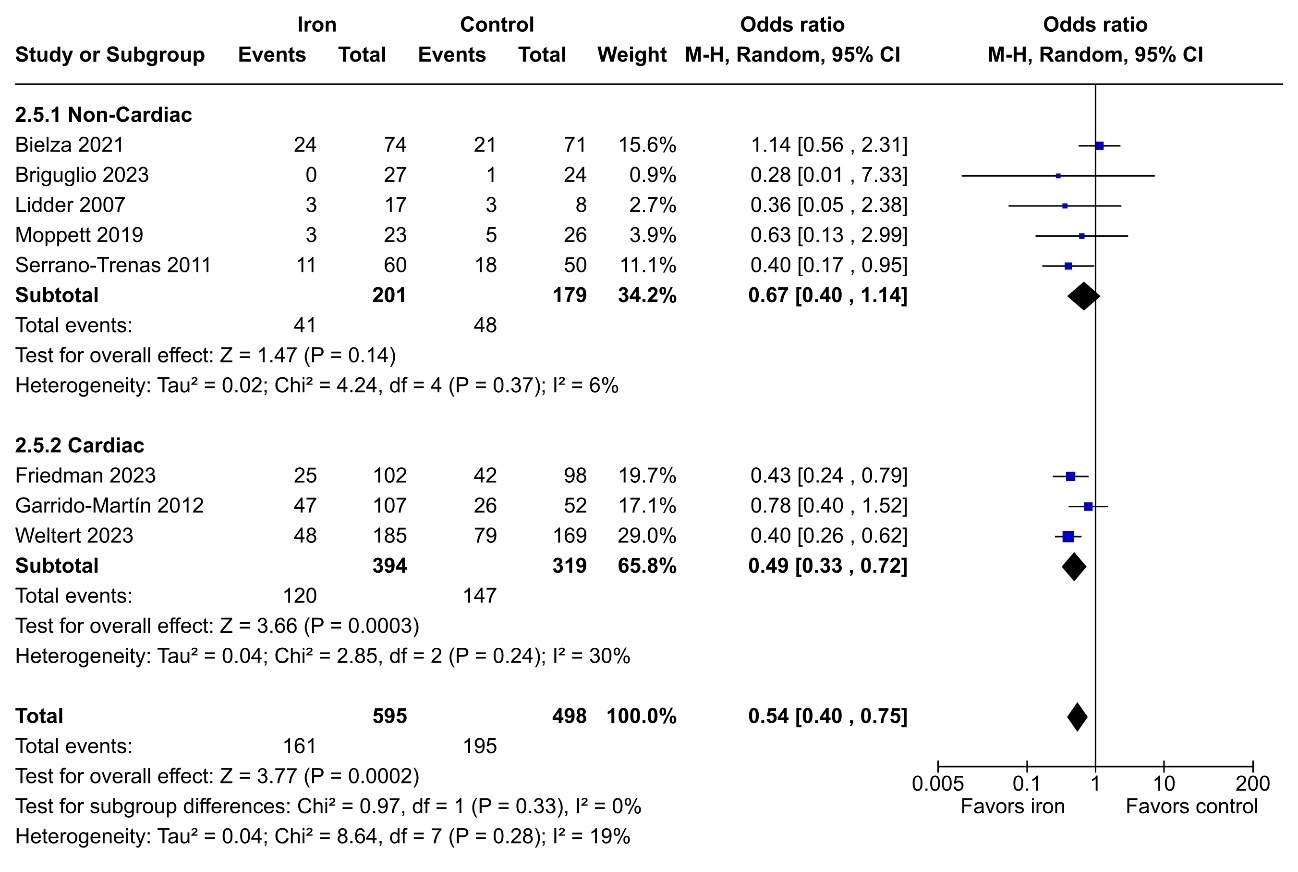


**Supplementary Figure S3** Trial sequential analysis of the primary outcome.


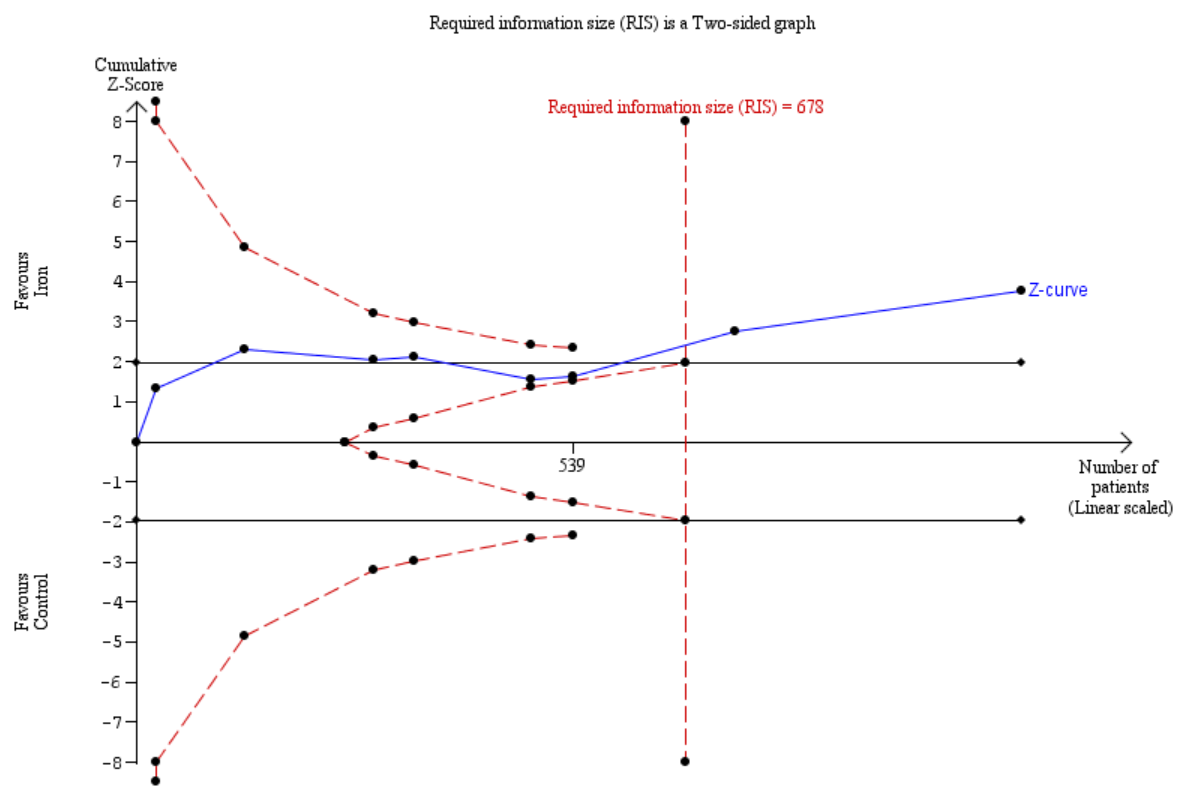


**Supplementary Figure S4** Trial sequential analysis of the primary outcome for the subgroup undergoing cardiac surgery.


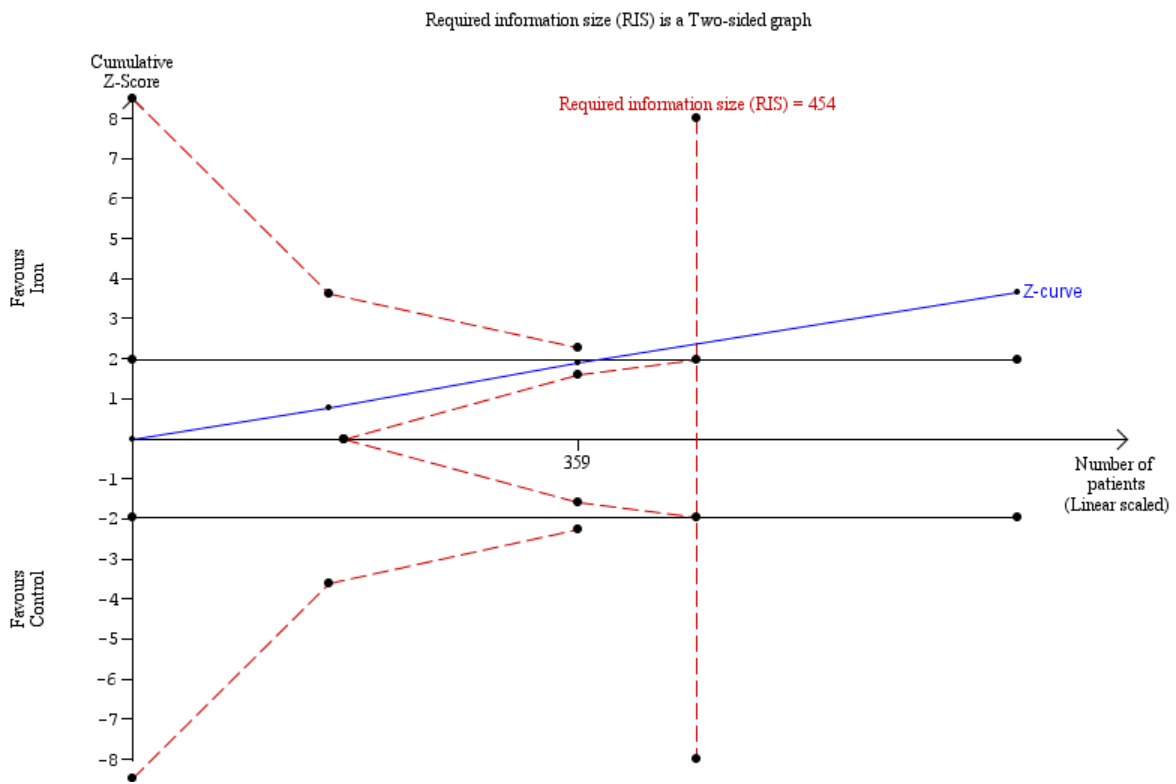


**Supplementary Figure S5** Trial sequential analysis of the primary outcome for the subgroup undergoing non-cardiac surgery.


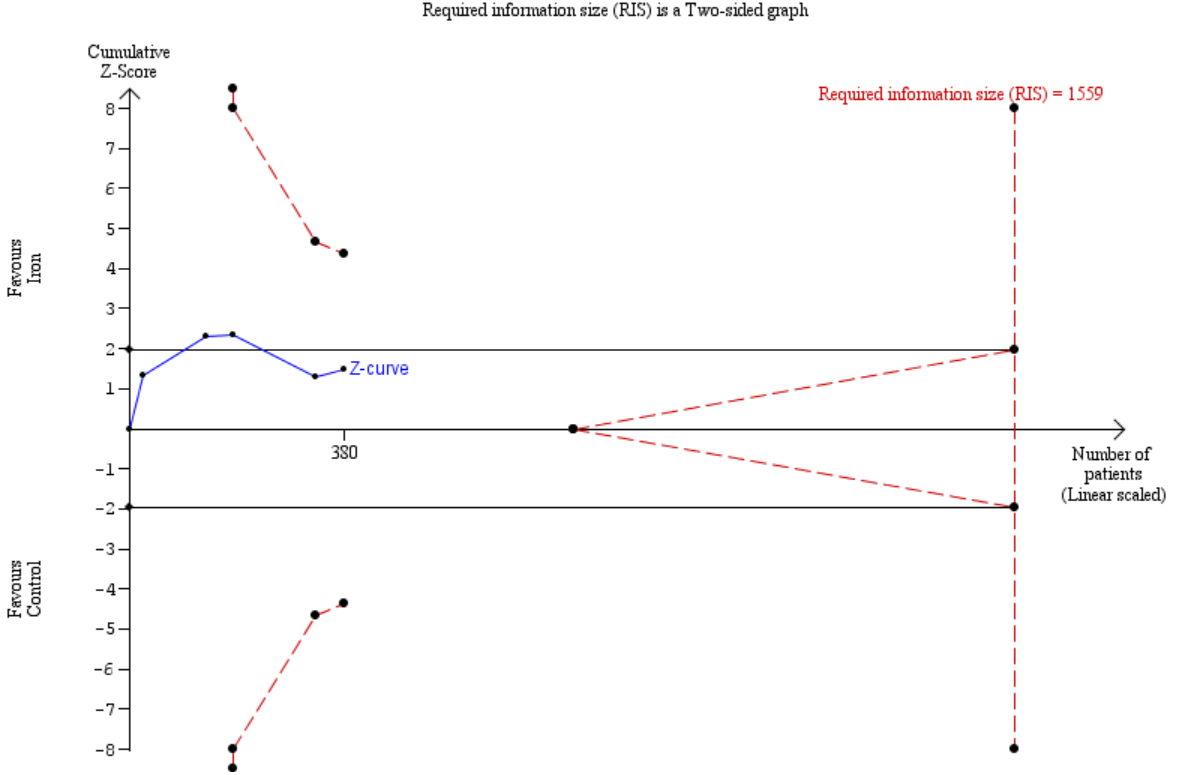


**Supplementary Figure S6** Meta-regression: Investigating, in the oral iron subgroup, the association between preoperative treatment duration and transfusion.

**Supplementary Figure S7** Oral subgroup leave-one-out analysis: Number of patients transfused.


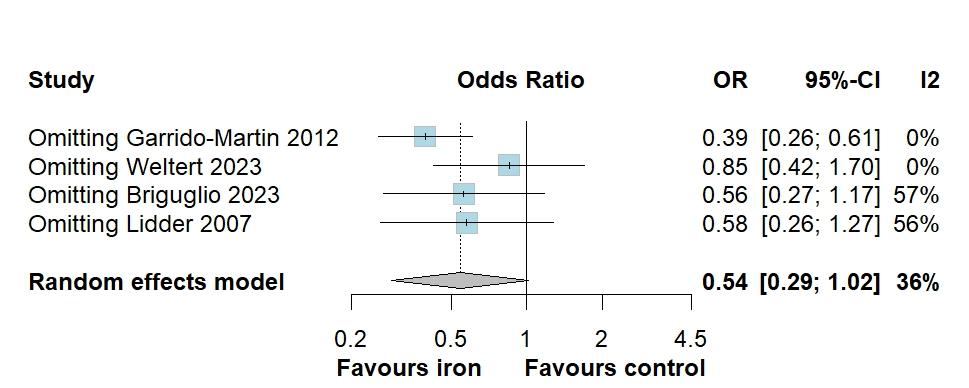


**Supplementary Figure S8** Quality assessment with the Cochrane tool for assessing risk of bias in randomized trials (RoB2).


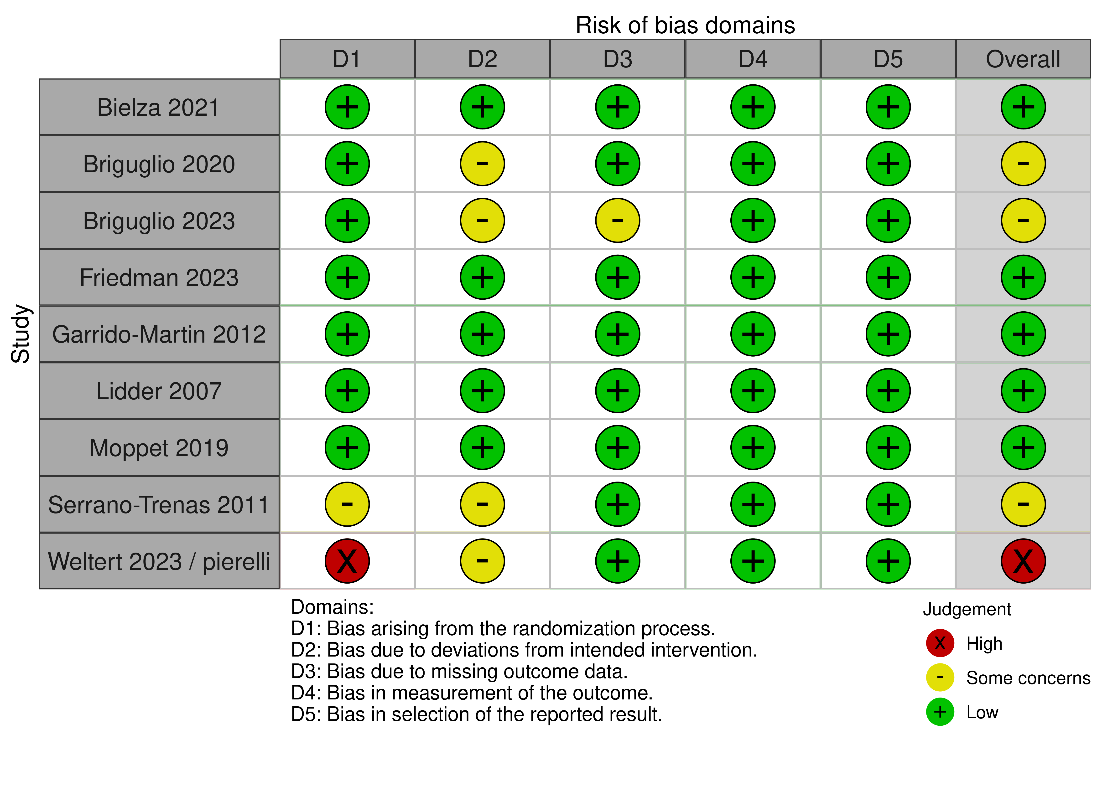


**Supplementary Figure S9** Funnel plot and Egger test: Number of patients transfused in total.
